# Report of a Case of Aneurismal Tumor Following Penetrating Wound of Shoulder

**Published:** 1872-06

**Authors:** C. C. F. Gay

**Affiliations:** Surgeon to the Buffalo General Hospital


ART. III.—*Report of a Case of Aneurismal tumor following penetrating wound of shoulder. Operation for ligation of right subclavian Artery.* By C. C. F. GAY, M. D. Surgeon to the Buffalo General Hospital.

On the morning of the 25th March 1872, William Dusenthal aged 22 years, was stabbed by a dirk knife, in the hands of one Donnaberger, while engaged in a general fight. The blade of the knife was six inches in length and three fourths of an inch in width and tapered to a point.

The young man bled profusely, and was without medical attendance for twenty-four hours, although physicians had been summoned. As nearly as I am able to judge, from the account given me by a member of the family (who speaks but indifferent English), the hemorrhage was twice suppressed by syncope; it was estimated that two quarts of blood had been lost, but was entirely controled when Dr. Burke arrived and took charge of the patient, and continued in daily attendance, administering stimulants tonics and nourishment.

On Friday morning April 5th, I was requested by Dr. Burke to visit the patient, along with him, in consultation. I was called on account of a pulsating tumor, that had first made its appearance upon, or near the shoulder, on the morning of April 3rd. On examination, I found the patient pale, pulse 120 per minute, troublesome cough, difficult respiration, bronchial rale, dullness on percussion over infraclavicular region, fingers numb, arm at times very painful—at intervals it felt dead, and the tumor excessively painful.

I applied stethoscope over the tumor, but no pressure could be made upon the tumor, on account of the intense pain the slightest pressure caused. All the symptoms of aneurism were readily detected, and there could be no possible doubt of the correctness of the diagnosis. The tumor was the size of the lower half section of an ordinary tea cup; but I believed it to be, but the presenting portion of an aneurism, the larger half, or more, of which, extended downwards behind the clavicle.

It occupied a position, midway between the neck and outer aspect of the shoulder, near or at the margin of the trapezius muscle, and about one inch back of the clavicle, upon the right side.

The wound made by the dirk was closed, and the cicatrix was three fourths of an inch in length, and just behind the clavicle.

It was believed that the subclavian artery been wounded, and that, as the pulsating tumor had so suddenly appeared, and was so rapidly enlarging, it would soon rupture and destroy the life of the patient by hemorrhage. I advised an operation, and Dr. Burke concurred with me, both in my diagnosis, and in the propriety of an operation. The patient begged of me to perfom it at once, although I had told him the danger that must attend it. On the following day Dr. Wetmore was called in consultation, who also concurred in the diagnosis, advised an operation at once, and assisted in its performance, in the presence of several medical gentlemen. Chloroform was administered and its effect maintained by ether. I cut down immediately upon the scalenus muscle, and exposed the artery to view. Its pulsations were so feeble as to be scarcely discernable by the eye or touch. At this stage of the operation not an ounce of blood had been lost. After sponging out the cavity dry, my eye rested upon a dark clot, upon which my finger was placed, when by its own weight it penetrated through, and into the pleural cavity. I could pass my fore finger its full length downward, upwards and laterally, without being able to measure the extent, of what I at first supposed to be an aneurismal sac. I discovered coagulated blood, extending from within the pleural cavity, upwards to the shoulder; scooping out upon my finger, just enough of the clot to show to the gentlemen present, that coagula was present. Upon withdrawing the finger fluid blood and two or three air bubbles escaped, but the orifice at once closed. At my request Dr. Wetmore now introduced his finger, and learned these facts, namely, that there had been internal hemorrhage, following the stabbing with the dirk knife, and the vein was now discovered, just at the orifice, from which the internal hemorrhage had proceeded, and so soon as I had broken up the coagula, this vein began to bleed. It was ligated at once, when all hemorrhage ceased. The presence of this large amount of coagulated blood within, and above, the pleural cavity, was an unpleasant discovery. Its presence was unexpected. It was there and had been there for days. Its presence was not at all desirable. It was unsought for and unwished for. It was, and is, beyond the wisdom of any man, however skillful, to have made a prior discovery of the presence, and extent of this coagula, by any diagnostic means at present in our possession. It might perhaps, have been wise then and there to have abandoned any further operation interference, since there, could have been but one conclusive logically reached, namely, the inevitably fatal result, sooner or later (and much sooner than later) under any circumstances. I however proceeded to, and did ligate the artery at its third portion. I also applied a second ligature, and as doubt was expressed as to the portion of the artery ligated, I dismiss this second ligature with this simple allusion to it, as being of no consequence whatever.

The operation lasted one hour. I should remark in passing, that when the integument and fascia along the clavicle were divided, the tumor presented the size, without exaggeration, of once and a half that of my closed hand, and so much impinged upon the space between it, and the clavicle, that there was but just room for my finger to pass between them. I forbore to make pressure upon it, or to crowd it back out of my way, fearing its walls might rupture. The location of the aneurism, namely, at the margin of the trapezius muscle, led to the supposition that the tranversalis colli, which terminates at this point, was the artery implicated; but the size of the tumor led to the belief that a much larger vessel was wounded and the locality pointed to the third portion of the Subclavian as the seat of injury, or that there might be given off from the third portion, a supernumerary artery, like a specimen I have in my possession, the artery being larger in this specimen, than any one of the branches of the thyroid axis.

"The wound was dressed and the patient placed in bed, there having been no subsequent hemorrhage. Twenty-four hours after the operation, the patient was visited by Dr. Burke and myself, and we congratulated ourselves and the patient, upon his comfortable condition. Pulsation in the tumor had entirely ceased, the general aspect and courage of the patient was good, although he still complained of numbness in his fingers. During the night he grew worse, and died at 4 o'clock the following morning.

Post Mortem by Dr. Briggs sixteen hours after death, in presence of several medical gentlemen. Upon opening the thoracic cavity, fluid and coagulated blood was found, estimated at one quart, within the right pleural cavity. It was in process of decomposition. The right lung at its apex was wounded, collapsed, and was undergoing decomposition; air had escaped from the wound of the lung and there was emphysema.

Upon carrying the dissections up to the shoulder, coagulated blood was found to extend to the aneurismal tumor; it was firmly impacted beneath and around it, imparting to the tumor, and lending to it size, that did not belong to it. By accidental pressure with my fore-finger, the sac burst, and the aneurismal clot was projected with considerable force into my hand. This clot was the size of a Bantam's egg, cupped at one extremity and coneshaped at the other extremity. It was hard, of a lightish color and mottled. In consequence of the rupture of the sac, and want of time, I have to regret that the dissections were not prosecuted to the extent of ascertaining the artery implicated in the aneurism.

I have thus given simply a narative of this case, which I regard as one of the most interesting and instructive surgical cases that it has ever been my province to observe; purposely reserving for a supplementory report, remarks upon some points, which I cannot but believe as of very great interest and value to the profession.

